# The Controversial Role of Adiponectin in Appetite Regulation of Animals

**DOI:** 10.3390/nu13103387

**Published:** 2021-09-26

**Authors:** Ni Tang, Xin Zhang, Defang Chen, Zhiqiong Li

**Affiliations:** Department of Aquaculture, College of Animal Science and Technology, Sichuan Agricultural University, Chengdu 61000, China; sautangni1992@163.com (N.T.); zhangxinscny@163.com (X.Z.); chendefang@sicau.edu (D.C.)

**Keywords:** adiponectin, appetite, food intake, macronutrient, fasting, nutrition, adipose tissue

## Abstract

Eating disorders and obesity are important health problems with a widespread global epidemic. Adiponectin (AdipoQ), the most abundant adipokine in the plasma, plays important roles in the regulation of energy homeostasis, glucose metabolism and lipid metabolism. Plasma adiponectin concentration is negatively associated with obesity and binge eating disorder. There is a growing interest in the appetite regulation function of adiponectin. However, the effect of AdipoQ on feeding behavior is controversial and closely related to nutritional status and food composition. In this review, we summarize the literatures about the discovery, structure, tissue distribution, receptors and regulation of nutritional status, and focus on the biological function of adiponectin in the regulation of food intake in the central and peripheral system.

## 1. Introduction

Eating disorders and obesity have become public health concerns that negatively affect the physical and mental health of human [1,2,3]. Understanding feeding behavior and corresponding regulation mechanism is beneficial to develop efficient therapeutic strategies. The desire to feed is mainly regulated by hormones secreted from brain such as Neuropeptide Y (NPY), Agouti Related Protein (AgRP), Cocaine- and Amphetamine-Regulated Transcript (CART), Proopiomelanocortin (POMC), Orexin and Nesfatin-1, and gastrointestinal tract such as Ghrelin, Cholecystokinin (CCK), Peptide YY (PYY) and Glucagon Like Peptide-1 (GLP-1) [4,5,6]. Peripheral tissues can perceive (sense) the changes of body energy metabolism, produce a series of appetite-related hormones and transmit appetite signals to the brain to regulate feeding behavior through nerve or endocrine pathways.

White adipose tissue (WAT) is not only a tissue for energy storage but also considered to be an endocrine organ, which can secrete a variety of hormones and cytokines [7]. Adiponectin (AdipoQ), mainly secreted from mammalian mature adipocyte, is the most abundant adipokine in the plasma. In the last decades, the functions of adiponectin in the regulation of insulin sensitivity [8,9,10], atherosclerosis [11], glucose uptake [12,13] and lipid metabolism [14,15] have been well established in vertebrates. In humans, there is growing evidence that eating disorders including anorexia nervosa and bulimia are associated with adiponectin expression [16,17,18,19]. However, few studies have investigated the role of adiponectin in appetite regulation in rodents [20,21,22]. It is worth nothing that the effect of AdipoQ on food intake is conflicting, including inhibiting food intake [20], promoting food intake [22] and not affecting food intake [23]. This discrepancy might be due to different nutritional status and dietary composition. Therefore, this review is aimed to summarize the function of AdipoQ on appetite regulation and its relationship with feeding status and nutrients.

## 2. Discovery

In 1995, Scherer et al., through random sequencing cDNA library on 3T3-L1 adipocytes, screened a new gene encoding protein of 30 kDa, whose structure was similar to that of complement factor C1q and was then called Adipocyte complement-related protein of 30 kDa (ACRP30) [24]. At the same time, three other research groups have isolated and identified adiponectin (AdipoQ) [25], gelatin-binding protein of 28 kDa (GBP28) [26] and Adipose Most Abundant Gene Transcript1 (APM1) [27] from humans and rodents, which were later identified as the same substance. It was not until 1999 that Arita et al. found that this gene was specifically expressed in human adipose tissue and then named adiponectin (AdipoQ) [28]. To date, *adipoq* gene has been discovered and identified from many vertebrates including mammals (e.g., mice [25], pigs [29] and rabbit [30]), birds (e.g., chickens [31], ducks [32] and geese [33]) and teleost fishes (zebrafish [34], rainbow trout [35], ayu [36] and large yellow croaker [37]). There is a lack of reports about adiponectin in amphibians and reptiles.

## 3. Structure

The structures of *adipoq* gene such as the chromosomal location, number of exons and encoding amino acid sequence were varied among species [24,31,34,35,38]. The *adipoq* gene of human, mammal (rat, pig, cow, sheep and rabbit) and fish (zebrafish) contains 3 exons and 2 introns, and the *adipoq* gene of bird (chicken) contains 2 exons and 1 intron. The number of amino acids encoded by fish *adipoq* was more than that of mammals and chicken (240–250 amino acids). Interestingly, two isoforms of *adipoq* genes (*adipoq-a* and *adipoq-b*) were identified from zebrafish genome database [34], while only one type of *adipoq* gene was identified from rainbow trout [35], ayu [36] and large yellow croaker [37].

**Figure 1:**
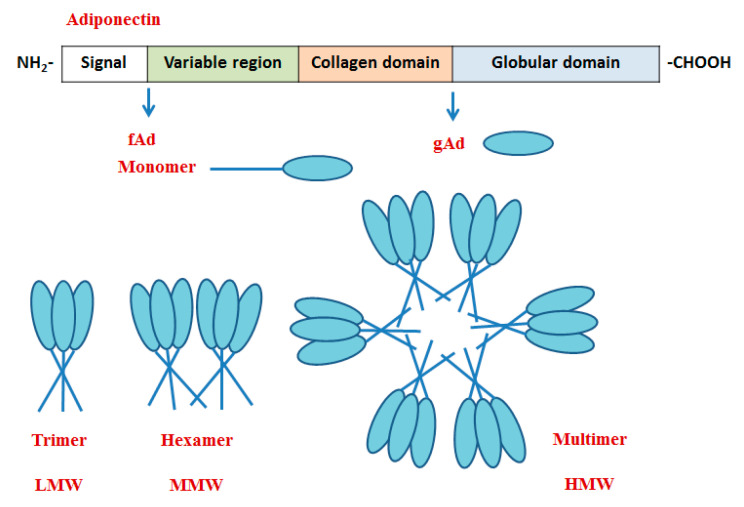
The structure of adiponectin. fAd, full-length adiponectin; gAd, globular domain adiponectin; LMW, low molecular weight; MMW, middle molecular weight; HMW, high molecular weight.

AdipoQ protein is consisted of four domains including N-terminal signal peptide, non-homologous region, collagen domain and C-terminal globular domain [24]. The collagen domain is composed of 22 Gly-X-Pro/Gly-X-X repeats, and the C-terminal spherical domain is similar to the structure of C1q and VIII type/X type collagen. The monomer of AdipoQ mainly exists in the form of full-length adiponectin (fAd) and globular domain adiponectin (gAd), which was cleavaged from fAd by leukocyte esterase [39]. Three complexes or multimers including low molecular weight (LMW, trimer), middle molecular weight (MMW, hexamer) and high molecular weight (HMW) were isolated and identified from human and mouse serum and by non-reducing and non-denatuated SDS-PAGE analysis [40]. The monomer of adiponectin forms a dimer by disulfide bond with the monomer, which polymerizes with a third monomer to form LMW adiponectin. Two homotrimers form MMW adiponectin by a disulfide bond (mouse N-terminal Cys39). Hexamers are linked by non-covalent bonds to form HMW adiponectin. The formation of the AdipoQ polymerization complex is regulated by hydroxylation and glycosylation of conserved lysine residues in the collagen domain (Figure 1) [41,42]. Studies on the function of AdipoQ have been focused on fAd, gAd, LMW, MMW and HMW AdipoQ, which mediated many signaling pathways with tissue specificity. For example, fAd, LMW, MMW and HMW activated the AMPK signaling pathway in the liver to inhibit gluconeogenesis, while gAd mainly promoted the phosphorylation of AMPK and ACC in skeletal muscle to promote fatty acid oxidation and glucose uptake [43]. Additionally, increasing studies have found that several peptide fragments located in globular domain mimic functions similar to AdipoQ [44,45,46,47,48].

## 4. Tissue Distribution

The mRNA of *adipoq* was widely distributed in vertebrates with different tissue distribution pattern. Initially, Maeda et al. found that *adipoq* was only specifically expressed in human adipose tissue, using northern blot analysis [26]. However, Chen et al. detected the expression of *adipoq* gene and protein in human placenta, especially syncytiotrophoblast cells, by using the real-time fluorescence quantitative polymerase chain reaction (RT-qPCR) method and western blot [49]. Recently, researchers have found that *adipoq* is also secreted locally by the eye [50]. Dai et al. used semi-quantitative reverse transcription and polymerase chain reaction (RT-PCR) to detect the distribution of *adipoq* mRNA in various tissues of pig, which was only detected in adipose tissue [38]. Taken together, *adipoq* is highly expressed not only in adipose tissue of mammals but is also detected in other tissues such as placenta and eyes.

Similar to mammals, *adipoq* gene is widely distributed in diverse tissues in birds with abundant expression in the adipose tissue. For example, in chicken, the mRNA expression of *adipoq* was highly expressed in adipose tissue, followed by liver, anterior pituitary, diencephalon, kidney and skeletal muscle [31]. Similarly, Yuan et al. also determined that *adipoq* mRNA was highly expressed in the adipose tissue, heart, stomach and skin of chicken, but was lowly expressed in the muscle [51]. In the chicken ovary, the adiponectin gene was found to be mainly expressed in theca layers and was suggested to exert paracrine or autocrine effects on ovarian steroidogenesis [52]. In chicken, western blot analysis showed that AdipoQ is secreted by theca layer cells isolated from chicken ovarian follicles [53].

At present, there are few studies on the distribution of *adipoq* in fish, and the only reports show that the tissue expression pattern of *adipoq* in fish is distinct from that of mammals and birds. In zebrafish (adult), *adipoq-a* mRNA was detected in the kidney; *adipoq-b* was mainly expressed in the brain and liver by semi-quantitative RT-PCR method [34]. In rainbow trout (juvenile), *adipoq* mRNA was abundantly expressed in red muscle and white muscle [54]. In ayu (juvenile), *adipoq* gene was highest expressed in the adipose tissue [36]. In large yellow croaker, *adipoq* was mainly expressed in the muscle [37], which did not mention the growth and development stage of the fish in this report.

In general, the current studies on the tissue distribution of *adipoq* mainly focus on mammals and birds, and there are few reports on other species. It is worth noting that *adipoq* expression in mammals and birds is mainly expressed in adipose tissue, while the abundant expression of *adipoq* in fish is not limited to adipose tissue. Whether and how AdipoQ is secreted by other tissues and organs remain to be explored.

## 5. Receptors

Adiponectin exerts various biological functions through binding to adiponectin receptors. Several adiponectin receptors have been identified, including adiponectin receptor 1 (AdipoR1), adiponectin receptor 2 (AdipoR2), T-cadherin (cadherin 13, cdh13) and Calreticulin (CRT). The structure of AdipoR1 and AdipoR2 contained 7 transmembrane domains with intracellular N-terminal and extracellular C-terminal, which is in contrast to G-protein-coupled receptors. The crystal structure of AdipoR1 and AdipoR2 has large gaps and 7 zinc binding sites within the transmembrane domains [55,56]. In mammals, AdipoR1 was mainly expressed in the muscle, while AdipoR2 was highly expressed in the liver [29,57]. In addition, AdipoR1 and AdipoR2 were expressed in the brain such as in hypothalamus, brainstem and cerebral cortex, in both human and rodents. In the hypothalamus, AdipoR1 and AdipoR2 are expressed by POMC and NPY neurons, which play critical roles in the regulation of feeding behavior [58]. These reports suggest that *adipoq* might have multiple biological functions via binding to AdipoR1 and AdipoR2.

As a member of the cadherin family, T-cadherin is a cell surface glycoprotein with glycosyl phosphatidylinositol (GPI) anchored and lacks transmembrane and cytoplasmic signaling domains [59]. T-cadherin is highly expressed mainly in endothelial cells and smooth muscle cells of mammals [60]. Numerous studies showed that T-cadherin, mainly expressed in the heart, muscle and aorta of mice, plays important roles in the accumulation of adiponectin in tissues [60,61]. In addition, T-cadherin is expressed by projection neurons within the main and accessory OBs in mice [62]. Immunofluorescence analysis showed that T-cadherin was co-localized with Type 2 TRC marker phospholipase C β2 in mice [63]. Additionally, T-cadherin is expressed in the brain of adult human, including cerebral cortex, medulla, thalamus as well as midbrain, by northern blot analysis. Whether T-cadherin mediates adiponectin function in the central nervous system is unclear.

Calreticulin is an endoplasmic reticulum (ER) luminal Ca^2+^-buffering chaperone [64]. Calreticulin mainly expressed on both the phagocyte and the apoptotic cell surface and was involved in adiponectin-mediated uptake of apoptotic cells. In human macrophages and THP-1 cells, the administration of anti-calreticulin antibody or small interfering RNA (siRNA) significantly blocked adiponectin-stimulated phagocytosis, which was not observed during treatment with AdipoR1, AdipoR2 or T-cadherin by siRNA method [65].

The affinity of adiponectin receptors to different forms of adiponectin varies in different tissues. AdipoR1 has a high affinity for gAd but a low affinity for fAd in the skeletal muscle, and AdipoR2 has moderate affinity for fAd and gAd in the liver [59]. T-cadherin works as a receptor for HMW and MMW but not for globular or LMW adiponectin. The adiponectin protein accumulates in several tissues, such as heart, and muscle through binding with T-cadherin [66]. It has been reported that adiponectin overexpression enhanced the regeneration of muscle in mice, but was not observed in T-cadherin null mice [67]. In differentiated C2C12 myotubes, the accumulation of adiponectin was suppressed when T-cadherin was knockdown. Recently, it has been shown that native adiponectin protein purified from mouse serum and HEK293 cells bind to cells expressing T-cadherin [68]. To date, a large number of studies have shown that AdipoR1 and AdipoR2 can mediate adiponectin to play a variety of biological roles. The functional studies of T-cadherin mainly focus on atherosclerosis, and whether T-cadherin has other biological functions remains to be explored.

## 6. The Effect of Feeding and Fasting on AdipoQ Expression

The nutritional status has shown to be an important modulator of adiponectin expression and secretion in the circulation and tissues. Studies on human reported that the concentration of AdipoQ in the blood is closely linked to the variants of adiponectin gene and appetite in human [69,70,71]. The change of AdipoQ level in the blood seems to serve as an indicator of appetite in humans. Numerous studies showed that patients suffering anorexia nervosa or bulimia nervosa have higher or lower adiponectin levels in the blood when compared with healthy individuals. A report on rodents has shown that the expression of AdipoQ can respond quickly to feeding. In mice, the levels of AdipoQ in the serum and cerebrospinal fluid were decreased at 3 h after feeding [22]. However, in obese mice, no significant difference of AdipoQ expression in serum and cerebrospinal fluid was observed before and after feeding [22]. These observations indicate that AdipoQ may act as a starvation signal that is quickly responsive to feeding in healthy mice.

The expressions of AdipoQ in the blood, adipose tissue, liver and muscle are affected by food restriction and food deprivation. Compared to rats fed ad libitum, short-term food restriction (3 days) did not alter body weight, adipose weight, *adipoq* gene expression in the adipose tissue and AdipoQ protein level in the serum of rat [72]. However, after long-term (30 days) restriction of feeding, the body weight and fat weight were significantly decreased and *adipoq* mRNA expression in white adipose tissue and AdipoQ protein expression in the serum were increased in rat [72]. Similar changes of *adipoq* in adipose tissue have been observed in teleost fish. In rainbow trout, fasting for 15 days and 35 days significantly increased *adipoq* mRNA expression in the adipose tissue [54]. These studies indicate that AdipoQ expression in the blood and adipose tissue was significantly elevated in response to prolonged food deprivation. Additionally, researchers examined adiponectin expression in other peripheral tissues such as liver and muscle after fasting for several days. In chicken, liver *adipoq* mRNA expression was significantly decreased after being fasted for two days [31]. In zebrafish, *adipoq-b* mRNA expression was decreased, after being fasted for four days, in the liver [34]. In rainbow trout, the expression of *adipoq* gene in the white muscle decreased significantly after being fasted for 15 days and 35 days [54]. These studies have shown that nutrient deficiency leads to an inhibitory effect on *adipoq* expression in the peripheral tissues (liver and muscle), which was related to the regulation of energy metabolism.

**Figure 2:**
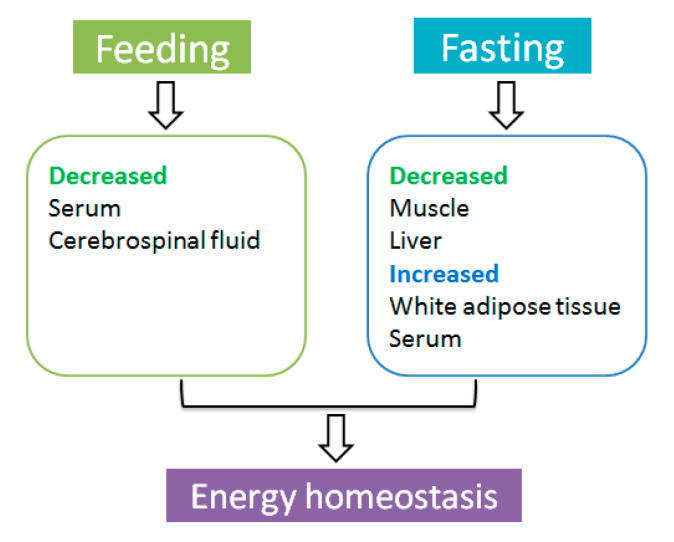
The effect of feeding and fasting on adiponectin expression.

Taken together, peripheral endocrine factor adiponectin acts as a messenger to perceive changes in nutritional status. Long-term energy deficiency resulted in a decline of *adipoq* mRNA expression in the liver and muscle to inhibit energy consumption, stimulating AdipoQ secreted from white adipose tissue into the blood to maintain energy homeostasis (Figure 2).

## 7. The Effect of Macronutrients on AdipoQ Expression

The food is mainly consisted of water and macronutrients, which is digested, absorbed and metabolized to meet biochemical utilization in the body. During feeding status, excess macronutrients are stored with the form of glycogen and triglycerides. In a state of fasting, glycogen and triglycerides are broken down into glucose and fatty acids, respectively, to meet energy requirements. This process is regulated by both nutrients and hormone to maintain energy homeostasis. Apart from the amount of the intake energy, the expression of adiponectin is greatly affected by macronutrients, which are a primary source for human and animals to generate energy to meet the needs for life [73,74,75].

In human studies, the proportion and type of carbohydrate are related to the concentration of adiponectin. High carbohydrate intake is one of the major reasons of induced metabolic syndrome and type 2 diabetes. Compared with healthy Japanese participants, the levels of total, LMW and HMW AdipoQ in serum were inversely associated with diabetes [76]. The participants consuming a higher carbohydrate (64% carbohydrate, 18% fat and 18% protein) for 6 weeks had lower concentration of AdipoQ in the blood, compared with a eucaloric moderate-fat diet (46% carbohydrate, 36% fat and 18% protein) [77]. Carbohydrate is composed of monosaccharides, disaccharides and polysaccharides. Diet enriched in fructose can induce insulin resistance and metabolic syndrome [78]. In overweight/obese adults, consuming fructose-sweetened beverages for 8 weeks decreased AdipoQ level in the plasma [79]. In obese women, non-alcoholic fatty liver disease (NAFLD) patients consuming sucrose foods were associated with lower adiponectin levels in the serum through a qualitative food frequency questionnaire [80]. In NAFLD patients, low molecular weight fucoidan and high stability fucoxanthin (LMF-HSFx) as a therapeutic approach treatment for 24 weeks increased AdipoQ expression [81]. Additionally, AdipoQ level is increased with dietary fiber intake [82]. The concentration of AdipoQ in the plasma was positively associated with total fiber intake and cereal fiber intake in diabetes-free women through a cross-sectional analysis [83]. To sum up, these studies suggest that obese people who eat a diet enriched in fructose or sucrose tend to have low level of adiponectin in the blood, and consuming several polysaccharides can raise blood AdipoQ level and alleviate symptoms of liver fibrosis.

The content and composition of dietary lipids were correlated with AdipoQ expression. Healthy postmenopausal women who were fed low-fat diets decreased AdipoQ expression in the plasma [84]. Compared with normal diet group, the expressions of *adipoq* mRNA and AdipoQ protein in the liver were significantly decreased when rat was fed with high fat diet for 16 weeks [85]. In addition, positive influence on adiponectin expression was observed in the presence of monounsaturated fatty acids and polyunsaturated omega-3 fatty acids. In individuals with cardiovascular risk factors, ω-3 supplementation showed an increase in serum AdipoQ [86]. A negative influence was found between AdipoQ expression and saturated fatty acids and trans-fatty acids. High saturated fatty acids intake may be associated with lower adiponectin levels in patients with type 1 diabetes mellitus (T1DM) [87].

Although many studies have reported that carbohydrates and lipids have effects on adiponectin expression, the information about the effect of protein and amino acids on adiponectin expression is little. Amino acids are general nutrients having anti-diabetic property. Glycine, one kind of essential amino acid, promoted the mRNA expression of *adipoq* in 3T3-L1 cells [88]. Additionally, amino acids (proline, phenylalanine and alanine) treatment increased *adipoq* mRNA and protein expression in human visceral adipocyte cells during high glucose environment (33 mM glucose) [89].

Based on the above studies, which examined the effect of macronutrients on adiponectin expression in human and rodents, we can conclude that the regulatory effect of carbohydrates and lipids depends on the content and the type of nutrients. However, the same is not clear about the effect of high- or low-protein dietary content on adiponectin expression. In addition, many studies have detected the mRNA level and total protein expression of adiponectin, but not active form of AdipoQ in circulation and tissues.

## 8. The Effect of AdipoQ on the Regulation of Appetite

### 8.1. The Anorexigenic Role of AdipoQ

#### 8.1.1. Central Effect

Several studies in rodents have demonstrated that AdipoQ treatment has an inhibitory effect on food intake. In mice (male, 8–10 weeks) fasted for 3 h, intra-cerebroventricular (*i.c.v*) injection of MMW AdipoQ (150 ng/g) significantly decreased accumulative food intake during 2 h, 3 h and 6 h after injection [21]. When Wistar rats (8 weeks) were food deprived for 6 h, *i.c.v* injection of recombinant AdipoQ significantly decreased cumulative food intake during 12 h [90]. These observations indicate that central AdipoQ administration can inhibit food intake under fasting status.

In the rat, the inhibition action of AdipoQ on food intake was reversed by administration of AdipoR1 specific antisense oligonucleotides but not AdipoR2 specific antisense oligonucleotide [90]. Moreover, *i.c.v* injection of AdipoQ immediately promoted the tyrosine phosphorylations of insulin receptor substrate 1 (IRS1), insulin receptor substrate 2 (IRS2) and extracellular regulated protein kinase (ERK), the serine phosphorylations of serine/threonine-protein kinase (Akt), forkhead box protein O1 (FOXO1), and tyrosine phosphorylations of janus kinase 2 (JAK2) and signal transducer and activator of transcription 3 (STAT3) in the hypothalamus, which were completely reversed after inhibition of AdipoR1. These studies suggest that AdipoQ inhibits food intake through acting on AdipoR1 and activating the signal transduction of IRS1/2-AKT-FOXO1 and JAK2-STAT3 signaling pathways in the hypothalamus of rodents.

**Figure 3:**
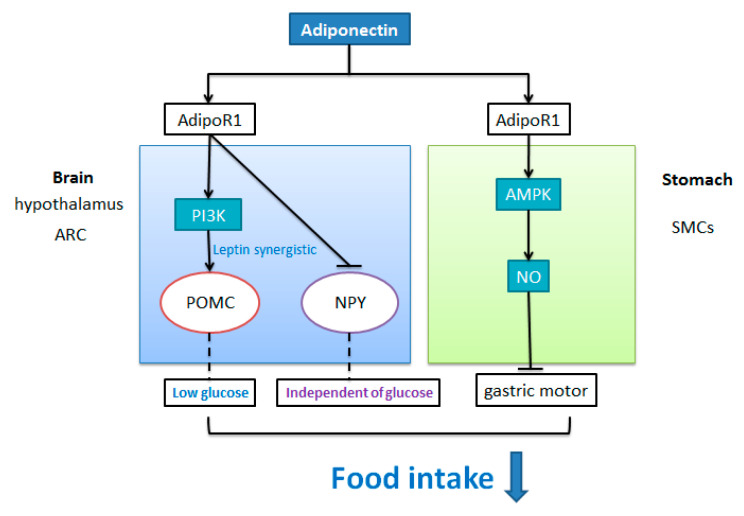
The inhibitory effect of adiponectin on food intake. In the arcuate nucleus of the hypothalamus, adiponectin may bind to AdipoR1 in the arcuate nucleus of the hypothalamus to stimulate the depolarization excitation of POMC neurons and inhibit the excitation of NPY/AgRP neurons, thus inhibiting food intake. The activation effect of AdipoQ on POMC neurons was closely related to low glucose concentration, PI3K and Leptin, but not to AMPK pathway. In addition, AdipoQ’s inhibitory effect on NPY neurons was independent of glucose concentration. Existing studies have in the peripheral confirmed that adiponectin can activate AMPK signaling pathway to promote NO release through binding with AdipoR1 in the basal part of the stomach, thus inhibiting food intake.

The receptors of AdipoQ including AdipoR1 and AdipoR2 were expressed by neurons (POMC and NPY neurons) in the rat hypothalamic nucleus [58]. In the hypothalamus arcuate nucleus (ARC), both POMC neuron and NPY/AgRP neuron project to other hypothalamic nuclei and then send projections to intrahypothalamic and extrahypothalamic regions to regulate appetite. The proopiomelanocortin (POMC)-expressing neuron, one of the key neurons in the brain, can be activated by nutritional and hormonal satiety signals to inhibit food intake of animals. Recent investigations on mice showed that AdipoQ significantly increased POMC neuronal membrane potential and the occurrence frequency of action potential through current clamp recording under a low concentration of glucose (5 mM) condition, which is to mimic fasted states [21]. Furthermore, the excitatory effect of AdipoQ on POMC neurons was reversed by phosphoinositol-3-kinase (PI3K) inhibitor [21,91] but not adenine monophosphate-activated protein kinase (AMPK) inhibitor [91]. Meanwhile, the effect of adiponectin on POMC neuronal activity was synergistically potentiated by leptin [91]. Sun et al. found that when leptin (100 nM) was co-incubated with AdipoQ (10 nM) or AdipoQ receptor agonist (AdipoRon, 5 μM) in mouse brain sections, the depolarization degree and action potential frequency of POMC neurons were significantly higher than that of AdipoQ administration [91]. Additionally, a previous study in mice showed that the effect of leptin on activation of POMC could also be blocked after inhibiting PI3K activity. These observations indicate that a synergistic effect might exist between AdipoQ and leptin in the regulation of POMC neurons. In summary, PI3K acts as a substrate for AdipoQ to inhibit food intake via acting on POMC neurons in rodents, and AdipoQ may cooperate with leptin to activate POMC neurons under a low concentration of glucose condition (Figure 3).

NPY/AgRP neuron in ARC is activated by hormonal and nutritional hunger signals to promote feeding. Simultaneously, adiponectin inhibits the orexigenic NPY/AgRP neurons. In vitro studies have shown that adiponectin can hyperpolarize mouse NPY neurons to inhibit their excitability, which could not be blocked by tetrodotoxin TTX administration [91]. Suyama et al. found that under the conditions of low concentration or high concentration of glucose, physiological dose of AdipoQ did not alter the resting membrane potential of ARC NPY neurons, but reduced the discharge of action potential in NPY neurons [21]. These observations suggest that AdipoQ can directly inhibited NPY/AgRP neurons independent of glucose concentration.

#### 8.1.2. Peripheral Effect

Besides the central inhibitory action of AdipoQ on feeding behavior, AdipoQ has been reported to exert a peripheral role in the regulation of food intake. Single peripheral injection (portal vein or intramuscular) of AdipoQ-encoding vector significantly decreased food intake (up to 280 day) and body weight in obese rat induced by high-fat diet [20]. Peripheral AdipoQ inhibits food intake of rodents under a state of excess energy.

The effect of AdipoQ on gastric motor responses might be a peripheral mechanism engaged by AdipoQ to regulate food intake. Idrizaj et al. reported that AdipoQ can influence the mechanical responses in strips from the mouse gastric fundus involving the nitric oxide (NO) pathway [92], which is a common target pathway involved in the hormonal regulation of gastrointestinal motility [93]. Furthermore, Idrizaj et al. observed an inhibitory action of AdipoQ on gastric smooth muscle cells (SMCs) excitation contraction coupling in mice through electrophysiological analysis [94]. Recently, Idrizaj et al. combined mechanical and electrophysiological methods, which further demonstrated that the effect of AdipoQ on K^+^ or Ca^2+^ current in SMCs was blocked in the presence of AMPK inhibitor (dorsomorphin). Meanwhile, western blotting analysis revealed that AdipoQ administration promoted the expression of phosphoAMPK (pAMPK) [95]. In addition, AdipoR1 was expressed by glial cells in the myenteric plexus, which was in keeping with the involvement of the AMPK pathway. These observations suggest that AdipoQ exerts an inhibitory action at the gastric smooth muscle via AMPK/NO pathway in the peripheral level, which might induce the decrease in food intake (Figure 3).

### 8.2. The Orexigenic Role of AdipoQ

#### 8.2.1. Central Effect

Few studies have demonstrated the stimulatory effect of AdipoQ on food intake in rodents. An early study by Kubota et al. showed that *i.c.v* administration of MMW adiponectin significantly increased 6 h food intake of mice, which was allowed to consume food for 3 h before injection [22]. Meanwhile, *i.c.v* injection of MMW adiponectin promoted the phosphorylation of AMPK and ACC in the ARH of mice [22]. The action of AdipoQ on food intake can be reversed by adeno-AdipoR1 siRNA or dominant-negative AMPK expression but not by AdipoR2 siRNA administration. Hypothalamic AMPK plays an important role in mediating hormonal regulation of feeding behavior [96]. This indicates that central AdipoQ through AdipoR1 activates AMPK signal pathway to promote food intake of mice under feeding status.

**Figure 4:**
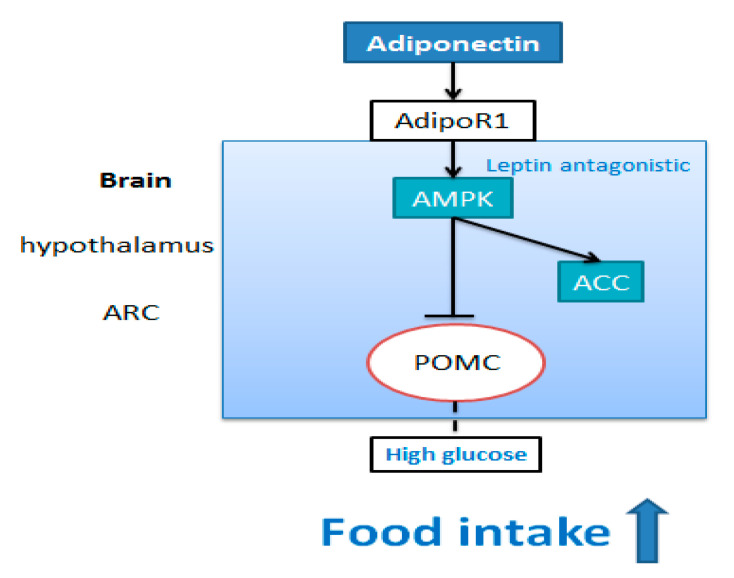
The stimulative effect of adiponectin on food intake. In the arcuate nucleus of the hypothalamus, adiponectin may bind to AdipoR1 in the arcuate nucleus of the hypothalamus to stimulate AMPK pathway and inhibit the excitation of POMC neurons. The inhibitory effect of AdipoQ on POMC neurons was closely related to high glucose concentration and AMPK pathway.

Suyama et al., using vivo and vitro experiments, revealed that the central effect of adiponectin on promoting food intake was associated with glucose conditions [21]. *I.c.v* injection of MMW AdipoQ and glucose significantly increased accumulative 6 h food intake after injection compared with single injection of glucose [21]. This result suggests that adiponectin increased food intake in the condition of high glucose in the brain. In the high glucose condition (10 mM), adiponectin directly decreased the frequency of cell membrane potential and action potential of POMC neurons of mice, which was reversed by AMPK inhibitor compound C. The above studies indicate that under the condition of high glucose (10mM), central AdipoQ may activate AMPK signaling pathway, inhibiting excitability of POMC neurons to increase food intake (Figure 4).

#### 8.2.2. Peripheral Effect

Intravenous injection (*i.v.*) of the full length of adiponectin (1 μg/g BW) significantly promoted accumulative 6 h food intake in mice under re-feeding status [22]. In mice, intravenous administration of leptin significantly inhibited food intake and decreased AMPK and ACC phosphorylation levels in arcuate nucleus of hypothalamus (ARH), while co-injection of full-length AdipoQ and leptin significantly reversed the inhibitory effect of leptin on food intake [22]. These results indicate that AdipoQ can reverse the inhibitory effect of leptin on AMPK signal pathway in the central nervous system. Furthermore, when AdipoR1 expression was inhibited by the administration of AdipoR1 siRNA, AdipoQ could not reverse the suppression of AMPK and acetyl-CoA carboxylaze (ACC) phosphorylation caused by leptin [22]. These results suggest that intravenous treatment of AdipoQ at least partly go across the blood–brain barrier acting on AMPK signal pathway in the arcuate nucleus of hypothalamus to promote food intake of rodents, which is the opposite of the action of leptin on food intake.

## 9. Conclusions

Adiponectin is a cytokine mainly secreted from adipose tissue in mammals, birds and a few fish species. Although adiponectin is mainly secreted from mammalian white adipose tissue, in non-mammalian vertebrates, the tissues secreting adiponectin may be diverse. The mRNA expression and protein expression of adiponectin in the circulation and tissues are affected by food intake and food deprivation. In addition, adiponectin protein expression in the blood has been shown to be affected by nutrients, especially the content and the type of carbohydrates and lipids. Additional studies are needed to explore the link between dietary protein and the expression of adiponectin.

Adiponectin (AdipoQ), one of the adipocytokines, is expected to be a new target for appetite regulation. To date, studies on AdipoQ’s regulation of animal feeding have only been conducted in rodents. The mechanism of AdipoQ regulating appetite mainly focuses on the central nervous system, especially the hypothalamus region. The control of appetite is not limited to the hypothalamus, but involves many other regions of the brain, especially the caudate stem, sensation and cortical-limbic system. It is unclear whether AdipoQ regulates food intake by acting on other brain regions. Additionally, the appetite regulation of AdipoQ seems mainly through AdipoR1, and whether other adiponectin receptors participate in AdipoQ appetite regulation remains to be studied.

The action of adiponectin as an appetite-stimulating factor or an appetite-inhibiting factor is controversial. The mechanisms of adiponectin regulating food intake are intricate and multifactorial, which are connected to feeding status, the content of glucose in the cerebrospinal fluid and the degree of fatness. Taken together, these results about appetite regulation of adiponectin suggest a potential therapeutic role for adiponectin in the treatment of eating disorders and obesity. As described above, adiponectin expression is tightly related to feeding status and nutrients. Future potential strategies to treat eating disorders related to abnormal expression of adiponectin should consider nutrients as a way to mediate the adiponectin signaling pathway.

## Data Availability

Not applicable.
